# Inter-observational analysis of computed tomography parameters to predict nonobvious posterior ligament complex injury in neurologically intact patients with thoracolumbar trauma

**DOI:** 10.1016/j.bas.2024.102855

**Published:** 2024-07-01

**Authors:** Joana Araújo de Azevedo, Carolina Garcez Martins, Nuno Oliveira, Pedro Varanda, Bruno Direito-Santos

**Affiliations:** a Department of Orthopedic Surgery of Hospital de Braga, Braga, Portugal; b School of Medicine, Minho University, Braga, Portugal

**Keywords:** Computed tomography, CT parameters, Magnetic resonance imaging, Posterior ligament complex, Spine trauma, Thoracolumbar fractures

## Abstract

**Introduction:**

Assessing the integrity of the posterior ligament complex (PLC), as a key element in the characterization of an unstable Thoracolumbar fracture (TLF), is challenging, but crucial in the choice of treatment.

**Research question:**

How to create a reproducible score using combined parameters of Computed Tomography (CT) to predict nonobvious PLC injury. How CT parameters relate with PLC status.

**Material and methods:**

Retrospective analysis of neurologically intact patients with an acute traumatic TLF, who underwent CT and Magnetic Resonance Imaging (MRI) within 72 h, in the Emergency Department of a single institution between January 2016 and 2022. Four investigators rated independently 11 parameters on CT and PLC integrity on MRI. The interrater reliability of the CT parameters was evaluated, and two risk scores were created to predict PLC injury on CT using the coefficients of the multivariate logistic regression.

**Results:**

154 patients were included, of which 62 with PLC injury. All CT measurements had excellent or good interrater reliability. Patients with Horizontal Fracture of the lamina or pedicle (HLPF), Spinous process fracture (SPF) and Interspinous Distance Widening (IDW) were positively associated with PLC injury (p < 0.001, p < 0.001 and p = 0.045, respectively). Risk Score 2 (RS2), which included only statistically significant variables, had a total of 75.9% of correct classifications (p < 0.001), with a sensitivity of 71.0% and specificity of 78.3% to estimate PLC injury detected in the MRI.

**Discussion and conclusion:**

Standardized procedures pre-established in the CT measurement protocol were effective. Identically to early findings, those three CT measurements showed a positive relation to PLC injury, thus enhancing the conclusions of previous studies. Comparing to the reliability of the CT findings above mentioned, the score was less precise.

## 1 Introduction

Worldwide incidence of traumatic spine fractures is reported to be 10.5 cases per 100,000 persons, with the lumbar spine segment being the most affected, followed by the thoracic and cervical segments (Alhadhoud and Alsiri, 2021; de Almeida Prado et al., 2021; Luna et al., 2017). Therefore, thoracolumbar fractures (TLFs) are among the most common spinal injuries in Emergency Departments (EDs).

TLFs can be associated with injuries with different severity levels (Trauma Programs, 2022). About 50% cause deformity, instability, or neurological deficits (Boos and Aebi, 2008; Ganjeifar et al., 2019).

Currently, the main decision-making factor in the therapeutic strategy is the assessment of fractured spine stability or the presence of neurological deficits (Ganjeifar et al., 2019; Khurana et al., 2019; Smith et al., 2010; Vaccaro et al., 2006). Controversy persists in those cases with normal neurologic status which could have an unfavorable, but preventable, evolution due to an insufficiently evaluated degree of instability.

The Posterior Ligament Complex (PLC) is one of the most important structures in stabilizing the spine (Maheswaran et al., 2020; Aly et al., 2021; van et al., 2013). When injured, it is associated with late spinal deformity, persistent pain and may also lead to neurological deficit (Maheswaran et al., 2020; Smith et al., 2010).

Magnetic Resonance Imaging (MRI) is currently the best imaging modality available to assess PLC integrity (Khurana et al., 2018). However, it is associated with higher costs and longer duration, and it is not available in all centers and countries equally. It can be challenging in patients with pacemakers or other incompatible devices, namely metallic implants, and is not feasible in polytraumatized patients, who often have associated multi-organ lesions and unstable hemodynamic status (Khurana et al., 2018).

Computed Tomography (CT) is a quick exam that overcomes some of the limitations inherent to MRI, being considered an integral part of trauma assessment protocol (Khurana et al., 2018; Pizones and Castillo, 2013; Hessmann et al., 2006). It is an accurate exam in the diagnosis of vertebral fractures, with a sensitivity of near 100 percent for the thoracolumbar spine, and widely considered the gold standard in the evaluation of bone structures (Sixta et al., 2012a). However, PLC injury is still difficult to assess on CT.

Although studies focusing on the combined analysis of parameters have already been carried out, they report limitations due to the small sample size, the non-objective definition of the measurement parameters, or the choice of inclusion and exclusion criteria (Ganjeifar et al., 2019; Khurana et al., 2018; Aly et al., 2022a).

Recent studies showed very good agreement between positive CT parameters and MRI to detect PLC injury. Aly et al. reported an independent positive relation between vertebral translation, facet joint malalignment, spinous process fracture, horizontal laminar fracture and interspinous widening with PLC injury on MRI (Aly et al., 2021, 2022b). In another paper, Aly et al. also demonstrated that using appropriate CT criteria, we will be able to correctly classify 90% of fractures comparing with MRI (Aly et al., 2023).

Our aim is to expedite the clinical decision by evaluating the diagnostic value of CT parameters and creating a reproducible score using combined CT parameters in the diagnosis of nonobvious PLC injury.

## 2 Methods

A retrospective analysis of all patients who underwent CT and MRI of the thoracic or lumbar spine at the ED of a single institution between January 2016 and 2022 was conducted. Inclusion criteria included history of acute thoracolumbar trauma, CT and MRI performed within 72 h, one acute vertebral body fracture, thoracic or lumbar (T1-L4), detected on CT and age greater than 18 years. We have included geriatric patients because they represent a substantial sample of patients evaluated in ED context. Exclusion criteria included neurological deficits, multiple vertebral body spine fractures, previous spine surgery, translation/rotation injuries, pathological fractures, inadequate coverage by MRI or CT images or motion artifacts in MRI. Patients with neurological deficits were excluded because, independently of the PLC status, they will need surgical treatment and ideally MRI study. Demographic and injury characteristic data (age, sex, mechanism of injury, number of vertebral body spine fractures, levels of the fractures and neurological status) were collected.

### 2.1 CT and MRI imaging Acquisition

CT images were acquired on a 64-slice CT scanner (Somatom go.All, Siemens Healthcare) in a helical mode using the following parameters: 120/220 kV/mA, radiation doses of 19.7 mGy and 607 mGy*cm, rotation time of 1.00 s, and a pitch of 0.80. The slice thickness for multiplanar reformatted images of bone algorithm was less than 1.5 mm. MRIs were performed on three different scanners: on a 3 T MRI scanner (Magnetom Skyra, Siemens Healthcare) and two 1,5 T MRI scanners (Magnetom Avanto, Siemens Healthcare and Achieva Pulsar, Philips Healthcare). The spine trauma protocol included sagittal T2-weighted and T1-weighted images, sagittal short tau inversion recovery (STIR) images and axial T2-weighted and T1-weighted images in the affected area.

### 2.2 Imaging analysis

Four investigators (JAA, CGM, NO, BDS) assessed CT and MRI images. After receiving consensus training to standardize the measurement and evaluation of each CT parameter, each investigator independently analyzed every CT image. During data collection, investigators were blinded to clinical information, original interpretation, and other readings. Discordant findings regarding MRI images were resolved by the most experienced spinal surgeon.

### 2.3 CT assessment of PLC injury

**Fig. 1:**
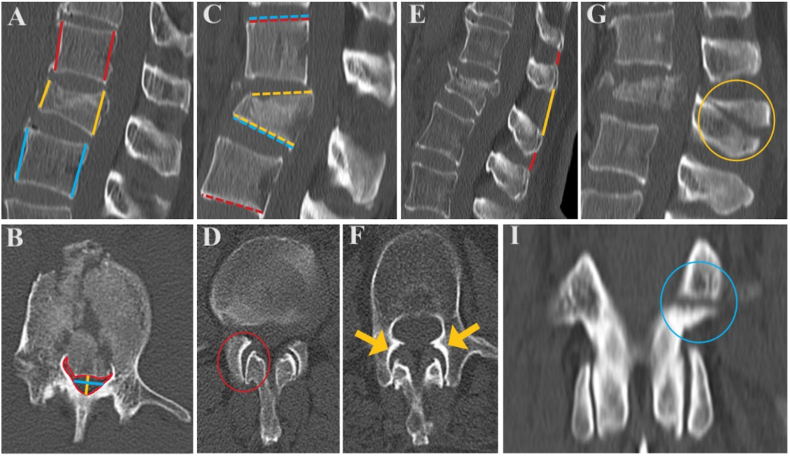
CT parameters used for diagnosis of PLC injury. CT sagittal images (A, C, E, G) showing (A) measurement of the UAVH and UPVH (red lines), AVH and PVH (yellow lines), LAVH and LPVH (blue lines); (C) measurement of LK (yellow dashed lines), RK (red dashed lines), GDA (blue dashed lines); (E) ISD >4 mm (yellow line) compared with adjacent levels (red lines); (G) a SPF (yellow circle). CT axial images (B, D, F) showing (B) measurement of the MSD (yellow line), TD (blue line) and direct measurement of TCA (red line); (D) FJW (red circle); (F) bilateral FJM (yellow arrows). CT coronal image (I) showing unilateral horizontal laminar fracture (blue circle). (For interpretation of the references to colour in this figure legend, the reader is referred to the Web version of this article.)

The following CT qualitative parameters were collected: avulsion, oblique or transverse fracture of the spinous process (SPF), facet joint malalignment (FJM), facet joint widening (FJW) and horizontal fracture of the lamina or pedicle (HLPF). The following quantitative CT parameters were measured: anterior vertebral body height (AVH), posterior vertebral body height (PVH), local kyphosis angle (LKA), regional kyphosis angle (RKA), Gardner's segmental deformity angle (GDA), mid-sagittal vertebral canal diameter (MSD), transverse vertebral canal diameter (TD), total canal cross-sectional area (TCA) and interspinous distance (ISD). Each CT parameter was assessed according to the measurement protocol elaborated (Table 1) (Fig. 1) (Khurana et al., 2019; Smith et al., 2010; Khurana et al., 2018; Bizdikian et al., 2021; Sixta et al., 2012b; OECD, 2015).

**Table 1:** CT parameters measurement protocol.

Parameter	Definition
AVH	The measurements are performed on the mid-sagittal CT image. The distance between the anterosuperior corner and anteroinferior corner of the vertebra is measured at three levels: at the fractured vertebra (AVH), at the upper adjacent vertebra (UAVH) and at the lower adjacent vertebra (LAVH). If osteophytosis is present, the measurements should avoid it.
UAVH	
LAVH	
PVH	The measurements are performed on the mid-sagittal CT image. The distance between the posterosuperior corner and posteroinferior corner of the vertebra is measured at three levels: at the fractured vertebra (PVH), at the upper adjacent vertebra (UPVH) and at the lower adjacent vertebra (LPVH). If osteophytosis is present, the measurements should avoid it.
UPVH	
LPVH	
ISD	The measurements are performed on the mid-sagittal image of the CT scan or on the image which better displays the spinous processes. The shorter distance between two adjacent spinous processes is measured at three levels: the biggest distance at the fractured vertebra level (ISD), the upper adjacent level (UISD) and the lower adjacent level (LISD).
UISD	
LISD	
LKA	The measurement is performed on the mid-sagittal image of the CT scan. A line is drawn along the superior and inferior endplates of the fractured vertebra. The angle formed between the lines is measured. Often the posterior aspect of the upper endplate has a ridge that distorts the normally flat surface of the body. In those cases, a line parallel to the flat surface of the body is drawn, ignoring the upper endplate ridge.
RKA	The measurement is performed on the mid-sagittal image of the CT scan. A line is drawn along the superior endplate of the upper adjacent vertebra and the inferior endplate of the lower adjacent vertebra. The angle formed between the lines is measured. Often the posterior aspect of the upper endplate has a ridge that distorts the normally flat surface of the body. In those cases, a line parallel to the flat surface of the body is drawn, ignoring the upper endplate ridge.
GDA	The measurement is performed on the mid-sagittal image of the CT scan. A line is drawn along the inferior endplate of the fractured vertebra and the superior endplate of the upper adjacent vertebra. The angle formed between the lines is measured. Often the posterior aspect of the upper endplate has a ridge that distorts the normally flat surface of the body. In those cases, a line parallel to the flat surface of the body is drawn, ignoring the upper endplate ridge.
SPF	The evaluation is performed on the mid-sagittal image of the CT scan. It is present when a transverse, avulsion or oblique fracture is observed in the spinous process, only at the level of the vertebral body fracture.
MSD	The measurements are performed on the axial CT image. The distance between the anterior canal border (defined as the posterior border of the mid-vertebral body) and posterior canal border (defined as the convergence of the superior margins of the laminae at the midline of the spinous process) is measure at three levels: at the fractured vertebra (MSD), at the upper adjacent vertebra (UMSD) and at the lower adjacent vertebra (LMSD). The measurements are performed on the axial CT image which correlates to the highest degree of vertebral body posterior retropulsion on the sagittal CT scan image.
UMSD	
LMSD	
TD	The measurement is performed on the axial CT image. The distance between the medial borders of the pedicles at the mid-pedicle level is measure at three levels: at the fractured vertebra (TD), at the upper adjacent vertebra (UTD) and at the lower adjacent vertebra (LTD). The measurements are performed on the axial CT image which correlates to the highest degree of vertebral body posterior retropulsion on the sagittal CT scan image.
UTD	
LTD	
TCA	The measurement is performed on the axial CT image. The total area of the canal is bordered: anteriorly by the posterior border of the vertebral body; posteriorly by the convergence of the superior border of the laminae at the midline of the spinous process; laterally by the medial border of the pedicles. It is measured using an electronic digitizer to outline the perimeter of the spinal canal and to calculate the precise total cross-sectional area at three levels: at the fractured vertebra (TCA), at the upper adjacent vertebra (UTCA) and at the lower adjacent vertebra (LTCA). The measurements are performed on the axial CT image which correlates to the highest degree of vertebral body posterior retropulsion on the sagittal CT scan image.
UTCA	
LTCA	
FJW	The measurement is performed on the axial CT image. The distance between facet(s), unilaterally or bilaterally, is measure.
FJM	The evaluation is performed on the axial CT image. It is present when, unilateral or bilateral, facet(s) are dislocated, perched, or showing a displaced fracture.
HLPF	The evaluation is performed on the coronal image of the CT scan. It is present when, unilateral or bilateral, horizontally oriented fracture(s) are observed in the lamina or pedicle.

*CT* Computed Tomography, *AVH* Anterior Vertebral Body Height, *UAVH* Upper Anterior Vertebral Body Height, *LAVH* Lower Anterior Vertebral Body Height, *PVH* Posterior Vertebral Body Height, *UPVH* Upper Posterior Vertebral Body Height, *LPVH* Lower Posterior Vertebral Body Height, *ISD* Interspinous Distance, *UISD* Upper Interspinous Distance, *LISD* Lower Interspinous Distance, *LKA* Local Kyphosis Angle, *RKA* Regional Kyphosis Angle, *GDA* Gardner's Segmental Deformity Angle, *SPF* Spinous Process Fracture, *MSD* Mid-Sagittal Vertebral Canal Diameter, *UMSD* Upper Mid-Sagittal Vertebral Canal Diameter, *LMSD* Lower Mid-Sagittal Vertebral Canal Diameter, *TD* Transverse Vertebral Canal Diameter, *UTD* Upper Transverse Vertebral Canal Diameter, *LTD* Lower Transverse Vertebral Canal Diameter, *TCA* Total Canal Cross-Sectional Area, *UTCA* Upper Total Canal Cross-Sectional Area, *LTCA* Lower Total Canal Cross-Sectional Area, *FJW* Facet Joint Widening, *FJM* Facet Joint Malalignment, *HLPF* Horizontal Fracture of the Lamina or Pedicle.

The following CT parameters were then calculated from the measurements previously mentioned: sagittal-to-transverse ratio (STR), percentage of canal occlusion (CO), percentage of anterior vertebral height loss (AVHL) and interspinous distance widening (IDW). The formulas used are present in Table 2 (Aly et al., 2021; Keynan et al., 2006; Mi et al., 2018; Jiang et al., 2018).

**Table 2:** CT parameters formulas protocol.

Parameter	Formula
STR	$\frac{MSD}{TD}$
CO	$\left[\frac{\frac{UTCA+LTCA}{2}-TCA}{\frac{UTCA+LTCA}{2}}\right]\times 100$
AVHL	$\left[\frac{\frac{UAVH+LAVH}{2}-AVH}{\frac{UAVH+LAVH}{2}}\right]\times 100$
IDW	$Present\;if:\left[ISD-\frac{UISD+LISD}{2}\right]\geq 4$

*CT* Computed Tomography, *STR* Sagittal-to-Transverse Ratio, *CO* Percentage of Canal Occlusion, *AVHL* Percentage of Anterior Vertebral Height Loss, *IDW* Interspinous Distance Widening, *MSD* Mid-Sagittal Vertebral Canal Diameter, *TD* Transverse Vertebral Canal Diameter, *UTCA* Upper Total Canal Cross-Sectional Area, *LTCA* Lower Total Canal Cross-Sectional Area, *TCA* Total Canal Cross-Sectional Area, *AVH* Anterior Vertebral Body Height, *UAVH* Upper Anterior Vertebral Body Height, *LAVH* Lower Anterior Vertebral Body Height, *ISD* Interspinous Distance, *UISD* Upper Interspinous Distance, *LISD* Lower Interspinous Distance.

### 2.4 MRI assessment of PLC injury

The PLC was assessed on MRI and classified as follows: no-disrupted, no MRI signal change or high signal due to ISL edema or facet joint effusion; or injured, identified by a discontinuity of the black stripe defining complete disruption of fibers of the SSL, FL, or ISL on T1-and T2-weighted images (Khurana et al., 2018).

### 2.5 Statistical analysis

Data was analyzed with SPSS®, version 26.0.

Comparisons between mechanisms of injury and age were performed with ANOVAs, complemented with Tukey HSD multiple comparisons tests.

The four investigators interrater reliability of CT assessments for continuous variables was analyzed with intraclass correlation coefficient (ICC) and with Fleiss kappa for categorical variables (Liljequist et al., 2019; Fleiss et al., 2003).

Comparisons between patients with no PLC injury and patients with PLC injury detected in MRI were performed with t-tests for continuous variables and with chi-square tests for categorical variables. Fisher exact test was used as an alternative to chi-square tests when Cochran's rules were not met.

Multivariate logistic regression was implemented to analyze the contribution of all consensus CT measurements, sex, and age, to detect PLC injury observed in MRI. Two risk scores based on the coefficients (β) of multivariate logistic regression were created. The first risk score (RS1) included all variables, the second (RS2) only the statistically significant variables. Quality of the risk scores was evaluated with ROC curves. We also calculated Cohen's K agreement measure between the PLC injury detected by the MRI and the estimate of each score. Statistical significance was set at p < .05.

## 3 Results

154 patients, 94 (61.0%) males and 60 (39.0%) females, with a mean age of 61.42 ± 16.27 (from 18 to 94 years), were enrolled in this study. 40.3% had PLC injury detected on MRI. The most prevalent fracture levels were L1 (38.3%), T12 (16.2%) and L2 (13.0%). The most prevalent mechanism of injury was fall from a height (59.7%) (Table 3).

**Table 3:** Sample characteristics.

	n (%)
Sex	
Female	60 (39.0%)
Male	94 (61.0%)
Levels of the fracture	
T2	3 (1.9%)
T3	4 (2.6%)
T4	1 (0.6%)
T6	4 (2.6%)
T8	2 (1.3%)
T9	5 (3.2%)
T10	5 (3.2%)
T11	8 (5.2%)
T12	25 (16.2%)
L1	59 (38.3%)
L2	20 (13.0%)
L3	9 (5.8%)
L4	9 (5.8%)
Mechanism of injury	
Fall from a height	92 (59.7%)
Ground level fall	39 (25.3%)
Motor vehicle accident	17 (11.0%)
Bicycle accident	4 (2.6%)
Dive	1 (0.6%)
Acts of violence	1 (0.6%)

n Absolute Frequency, % Relative Frequency.

Mechanism of injury was found to be associated with age (p < .001). Younger patients were associated with motor vehicle accidents (mean age of 50.53 ± 16.09) and older patients with fall from a height (mean age of 61.28 ± 15.23). Ground level fall was associated with the highest mean of age (69.64 ± 13.51).

For continuous variables, almost all measures had ICC higher than 0.90, therefore excellent interrater reliability. For categorical binary variables, results for FJM (0.649), HLPF (0.716) and IDW (0.729) were between 0.40 and 0.74 (intermediate to good) and >0.75 (excellent) for SPF (0.859). Consensus was established by the most experienced spinal surgeon (investigator 3) (Table 4).

**Table 4:** Descriptive statistics for CT measurements stratified by investigator and interrater reliability analysis.

Measurement	Investigator				ICC
	1	2	3	4	
UAVH	21.83 (3.35)	21.81 (3.34)	22.16 (3.35)	21.49 (3.17)	0.973
AVH	17.41 (4.49)	17.75 (4.66)	17.98 (4.61)	17.29 (4.55)	0.976
LAVH	24.23 (3.55)	24.18 (3.41)	24.59 (3.52)	23.79 (3.39)	0.966
UPVH	23.66 (3.26)	24.22 (3.23)	24.13 (3.38)	23.41 (3.24)	0.964
PVH	21.96 (3.38)	22.58 (3.44)	22.75 (3.37)	21.86 (3.42)	0.954
LPVH	24.99 (3.04)	25.53 (3.03)	25.56 (3.22)	24.70 (3.06)	0.956
UISD	10.55 (4.87)	10.66 (4.71)	10.03 (4.68)	10.52 (4.61)	0.965
ISD	10.58 (5.60)	10.85 (5.50)	10.20 (5.53)	10.82 (5.65)	0.976
LISD	10.08 (5.29)	10.25 (5.12)	9.72 (4.98)	10.39 (5.23)	0.971
LKA	9.23 (6.93)	9.01 (7.09)	9.24 (7.13)	8.70 (6.83)	0.954
RKA	5.54 (12.42)	5.55 (12.63)	6.12 (12.46)	5.38 (12.50)	0.983
GDA	9.82 (8.70)	10.10 (8.60)	10.21 (8.64)	9.25 (8.77)	0.970
SPF					
No	112 (72.7%)	119 (77.3%)	118 (76.6%)	117 (76.0%)	0.859 ^(a)^
Yes	42 (27.3%)	35 (22.7%)	36 (23.4%)	37 (24.0%)	
UMSD	17.37 (2.08)	17.30 (1.93)	16.95 (2.00)	16.90 (1.97)	0.921
MSD	15.01 (3.64)	14.88 (3.54)	14.96 (3.64)	14.46 (3.60)	0.968
LMSD	17.80 (2.82)	17.60 (2.65)	17.50 (2.79)	17.31 (2.79)	0.951
UTD	21.85 (3.54)	21.94 (3.50)	21.60 (3.50)	21.29 (3.54)	0.971
TD	22.63 (3.84)	22.74 (3.82)	22.46 (3.70)	22.01 (3.63)	0.970
LTD	23.34 (3.67)	23.42 (3.56)	23.01 (3.51)	22.84 (3.50)	0.972
UTCA	268.37 (58.06)	271.92 (58.32)	264.43 (59.03)	263.36 (58.49)	0.976
TCA	230.96 (62.85)	233.48 (63.53)	226.25 (63.91)	225.59 (63.77)	0.973
LTCA	282.44 (62.84)	284.49 (60.91)	277.80 (61.66)	277.67 (59.69)	0.971
FJW	2.29 (1.00)	2.28 (0.98)	1.79 (0.97)	1.84 (1.01)	0.812
FJM					
No	137 (89.0%)	135 (87.7%)	146 (94.8%)	145 (94.2%)	0.649 ^(a)^
Yes	17 (11.0%)	19 (12.3%)	8 (5.2%)	9 (5.8%)	
HLPF					
No	131 (85.1%)	113 (73.4%)	119 (77.3%)	128 (83.1%)	0.716 ^(a)^
Yes	23 (14.9%)	41 (26.6%)	35 (22.7%)	26 (16.9%)	
IDW					
No	88 (57.1%)	91 (59.1%)	84 (54.5%)	100 (64.9%)	0.729 ^(a)^
Yes	66 (42.9%)	63 (40.9%)	70 (45.5%)	54 (35.1%)	

*CT* Computed Tomography, *UAVH* Upper Anterior Vertebral Body Height, *AVH* Anterior Vertebral Body Height, *LAVH* Lower Anterior Vertebral Body Height, *UPVH* Upper Posterior Vertebral Body Height, *PVH* Posterior Vertebral Body Height, *LPVH* Lower Posterior Vertebral Body Height, *UISD* Upper Interspinous Distance, *ISD* Interspinous Distance, *LISD* Lower Interspinous Distance, *LKA* Local Kyphosis Angle, *RKA* Regional Kyphosis Angle, *GDA* Gardner's Segmental Deformity Angle, *SPF* Spinous Process Fracture, *UMSD* Upper Mid-Sagittal Vertebral Canal Diameter, *MSD* Mid-Sagittal Vertebral Canal Diameter, *LMSD* Lower Mid-Sagittal Vertebral Canal Diameter, *UTD* Upper Transverse Vertebral Canal Diameter, *TD* Transverse Vertebral Canal Diameter, *LTD* Lower Transverse Vertebral Canal Diameter, *UTCA* Upper Total Canal Cross-Sectional Area, *TCA* Total Canal Cross-Sectional Area, *LTCA* Lower Total Canal Cross-Sectional Area, *FJW* Facet Joint Widening, *FJM* Facet Joint Malalignment, *HLPF* Horizontal Fracture of the Lamina or Pedicle, IDW Interspinous Distance Widening. Results presented as means (M) and standard deviations (SD) for continuous variables, frequencies (n) and percentages (%) for categorical variables; Intraclass correlation coefficient (ICC) calculated to assess interrater observer reliability for continuous variables; (a)Fleiss Kappa agreement calculated to assess interrater observer reliability for categorical variables.

When comparing consensus measures by PLC injury detected in MRI, significant associations were found for SPF and HLPF (p < 0.001). Patients with PLC injury had higher prevalence of SPF (41.9% vs. 10.9%) and HLPF (45.2% vs. 7.6%) (Table 5).

**Table 5:** Consensus measures compared by PLC injury detected in MRI.

Consensus measures	Total	No PLC injury	PLC injury	Statistical test
LKA	9.24 (7.13)	8.75 (6.64)	9.97 (7.81)	t_(152)_ = -1.04, p = .302, d = −0.17
RKA	6.12 (12.46)	5.36 (12.42)	7.26 (12.54)	t_(152)_ = -0.93, p = .355, d = −0.15
GDA	10.21 (8.64)	9.92 (8.24)	10.65 (9.24)	t_(152)_ = -0.52, p = .605, d = −0.09
SPF				
No	118 (76.6%)	82 (89.1%)	36 (58.1%)	$\chi^{2}$_**(1)**_**=19.96, p<.001, φ=0.36**
Yes	36 (23.4%)	10 (10.9%)	26 (41.9%)	
FJW	1.79 (0.97)	1.74 (0.94)	1.87 (1.00)	t_(152)_ = -0.82, p = .415, d = −0.13
FJM				
No	146 (94.8%)	90 (97.8%)	56 (90.3%)	p = 0.061, φ = 0.18 _(b)_
Yes	8 (5.2%)	2 (2.2%)	6 (9.7%)	
HLPF				
No	119 (77.3%)	85 (92.4%)	34 (54.8%)	$\chi^{2}$_**(1)**_**=29.74, p<.001, φ=0.44**
Yes	35 (22.7%)	7 (7.6%)	28 (45.2%)	
IDW				
No	84 (54.5%)	52 (56.5%)	32 (51.6%)	$\chi^{2}$_(1)_ = 0.36, p = 0.549, φ = 0.05
Yes	70 (45.5%)	40 (43.5%)	30 (48.4%)	
STR	0.69 (0.20)	0.71 (0.18)	0.65 (0.22)	t_(152)_ = 1.65, p = .101, d = 0.27
CO	15.46 (20.25)	14.70 (17.72)	16.58 (23.63)	t_(152)_ = -0.57, p = .573, d = −0.09
AVHL	22.32 (19.98)	20.53 (18.89)	24.99 (21.37)	t_(152)_ = -1.37, p = .174, d = −0.22

*PLC* Posterior Ligament Complex, *MRI* Magnetic Resonance Imaging, *LKA* Local Kyphosis Angle, *RKA* Regional Kyphosis Angle, *GDA* Gardner's Segmental Deformity Angle, *SPF* Spinous Process Fracture, *FJW* Facet Joint Widening, *FJM* Facet Joint Malalignment, *HLPF* Horizontal Fracture of the Lamina or Pedicle, *IDW* Interspinous Distance Widening, *STR* Sagittal-to-Transverse Ratio, *CO* Percentage of Canal Occlusion, *AVHL* Percentage of Anterior Vertebral Height Loss.

Results presented as means (M) and standard deviations (SD) for continuous variables, frequencies (n) and percentages (%) for categorical variables; t-tests (t) and chi-square tests (${\;\chi }^{2}$) performed to compare continuous and categorical variables, respectively; Cohen's d (d) and phi (φ) to assess t-tests and chi-square tests effect size, respectively; (b) Fisher exact test calculated as alternative to chi-square test when Cochran rules were not met.

From our multivariate logistic regression model, we found that younger patients are less likely to have PLC injury detected in MRI, estimating 3% less odds for each year of age (p = 0.032). Patients with HLPF and SPF positive are more likely to have PLC injury detected in MRI (p < 0.001). Lastly, patients with IDW positive have 2.33 more odds of PLC injury detected in MRI (p = 0.045) (Table 6).

**Table 6:** Multivariate logistic regression adjusted for all consensus measurements, sex, and age for outcome PLC injury detected in MRI.

	β	S.E.	aOR	95% CI for aOR		p-value
				Lower bound	Upper bound	
Sex (male)	−0.01	0.46	0.99	0.40	2.43	0.985
Age	**−0.03**	**0.01**	**0.97**	**0.94**	**0.99**	**0.032**
LKA	0.06	0.07	1.06	0.93	1.21	0.365
RKA	0.01	0.03	1.01	0.94	1.08	0.779
GDA	−0.07	0.06	0.94	0.83	1.06	0.286
SPF (yes)	**1.81**	**0.53**	**6.08**	**2.15**	**17.20**	**<0.001**
FJW	0.15	0.28	1.17	0.68	2.00	0.580
FJM (yes)	1.05	1.15	2.86	0.30	27.26	0.360
HLPF (yes)	**2.04**	**0.58**	**7.66**	**2.47**	**23.76**	**<0.001**
IDW (yes)	**0.85**	**0.42**	**2.33**	**1.02**	**5.35**	**0.045**
STR	0.14	1.60	1.15	0.05	26.53	0.931
CO	−0.01	0.01	0.99	0.97	1.02	0.640
AVHL	−0.001	0.02	1.00	0.97	1.03	0.977

*PLC* Posterior Ligament Complex, *MRI* Magnetic Resonance Imaging, *LKA* Local Kyphosis Angle, *RKA* Regional Kyphosis Angle, *GDA* Gardner's Segmental Deformity Angle, *SPF* Spinous Process Fracture, *FJW* Facet Joint Widening, *FJM* Facet Joint Malalignment, *HLPF* Horizontal Fracture of the Lamina or Pedicle, *IDW* Interspinous Distance Widening, *STR* Sagittal-to-Transverse Ratio, *CO* Percentage of Canal Occlusion, *AVHL* Percentage of Anterior Vertebral Height Loss.

Multivariate logistic regression was implemented to analyze the contribute of all consensus CT measurements, sex, and age, to detect PLC lesion observed in MRI; β - beta coefficients of multivariate logistic regression; S.E. - standard error; adjusted odds ratios (aOR) and 95% confidence intervals (95% CI) were calculated to assess the effect size.

**Fig. 2:**
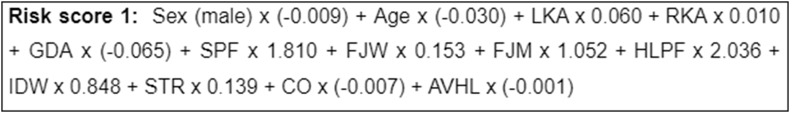
Risk score 1 LKA Local Kyphosis Angle, *RKA* Regional Kyphosis Angle, *GDA* Gardner's Segmental Deformity Angle, *SPF* Spinous Process Fracture, *FJW* Facet Joint Widening, *FJM* Facet Joint Malalignment, *HLPF* Horizontal Fracture of the Lamina or Pedicle, *IDW* Interspinous Distance Widening, *STR* Sagittal-to-Transverse Ratio, *CO* Percentage of Canal Occlusion, *AVHL* Percentage of Anterior Vertebral Height Loss.

**Fig. 3:**
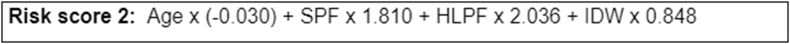
Risk score 2 *SPF* Spinous Process Fracture, *HLPF* Horizontal Fracture of the Lamina or Pedicle, *IDW* Interspinous Distance Widening.

Two risk scores were then calculated. ROC curves analysis demonstrated that, for RS1 (Fig. 2), a patient's result above −0.3995 has a sensitivity of 72.6% and specificity of 77.2%, with an area under the curve (AUC) of 82.5%. For RS2 (Fig. 3), a patient's result above −0.6500 has a sensitivity of 71.0% and specificity of 78.3% with an AUC of 80.8%.

When studying the association of estimated classifications of PLC injury detected by the risk scores and PLC injury detected in MRI, both risk scores yielded similar results, with a total of 75.9% of correct classifications (p < 0.001). A moderate agreement of 0.483 was assessed with Cohen's K. These results show that RS2 is more parsimony than RS1, because with a set of four independent variables, agreement was equal to RS1, despite slightly lower AUC results (Table 7).

**Table 7:** Association of estimated classifications of PLC injury detected by risk scores and PLC injury detected in MRI.

	PLC injury detected in MRI		Statistical test
PLC injury estimated by risk score 1	No	Yes	
No	80 (51.9%)	25 (16.2%)	$\chi^{2}$_**(1)**_**=37.13, p<.001, φ=0.49**
Yes	12 (7.8%)	37 (24.0%)	Cohen's K = 0.483

PLC injury estimated by risk score 2			
No	80 (51.9%)	25 (16.2%)	$\chi^{2}$_**(1)**_**=37.13, p<.001, φ=0.49**
Yes	12 (7.8%)	37 (24.0%)	Cohen's K = 0.483

*PLC* Posterior Ligament Complex, *MRI* Magnetic Resonance Imaging.

Results presented as frequencies (n) and percentages (%) overall; chi-square tests (${\;\chi }^{2}$) performed to compare categorical variables; phi (φ) to assess chi-square tests effect size; Cohen's K to assess interrater reliability.

## 4 Discussion

Despite many attempts to obtain more reliable methods, the diagnosis of spinal instability is still considered challenging in the clinical scenario (Ganjeifar et al., 2019). However, PLC integrity has proven to be a determinant element of spine stability, being increasingly used to determine an appropriate treatment strategy for TLFs (Vaccaro et al., 2006; Khurana et al., 2018).

In our study, 40.3% of patients had PLC injury detected on MRI. Although being an imperfect reference standard, MRI shows high sensitivity (79–100%) and specificity (53.5–100%) for PCL injury detection, second only to intraoperative findings (Khurana et al., 2018). We selected patients with CT and MRI performed within 72 h, to attempt preserving MRI sensitivity (Aly et al., 2022b). The well-defined eligibility criteria allowed us to exclude those with high pretest probability of PLC injury (neurological deficits and rotation/translation injuries), so we managed to focus on nonobvious PLC injuries, enhancing the relevance of our results for decision making (Aly et al., 2021; Costachescu et al., 2022).

Study participants were mainly males and middle-aged. The most prevalent levels of fracture were L1, followed by T12 and L2, therefore, consistent with literature, which identifies the thoracolumbar junction as the most affected by trauma (Alhadhoud and Alsiri, 2021; Costachescu et al., 2022). The most prevalent mechanism of injury was fall from a height. The youngest patients were associated with motor vehicle accidents, the oldest with ground level falls. We also found that younger patients were less likely to have PLC injury detected in MRI. Owing to the mechanism of injury, the latter may appear contradictory, since the youngest group of patients is associated with injuries that apparently result from more violent trauma. However, as previously mentioned, we excluded the most concerning cases, explaining less prevalence of PLC injury in this sample subgroup. Unlike prior studies, we included older patients, considering the growing relevance of low-energy falls in the elderly population due to global aging (Khurana et al., 2018; Aly et al., 2022a; Tins, 2017).

Involving four investigators with different levels of experience in CT reading enhanced result reproducibility. However, by standardizing the methodology of CT measurements, with a consistent definition for each individual CT parameter, we managed to obtain very good to excellent interrater reliability, suggesting the effectiveness of pre-established procedures.

FJM and HLPF had the lowest interrater reliability, even though they were considered intermediate to good. That can be explained by the lack of consensus in literature to define the degree of facet fracture displacement needed to report FJM as positive, which influenced our definition of this parameter, and also by the commonly misinterpretation of HLPF as vertical fracture of the lamina or pedicle (VLPF) (Aly et al., 2021).

The main focus of our study was to evaluate eleven CT parameters to assess their potential association with PLC injury. To the best of our knowledge, this is the first study to analyze these many CT parameters while combining predominantly excellent results in interrater reliability of four different raters (Ganjeifar et al., 2019; Aly et al., 2021, 2022a; Khurana et al., 2018; Aly et al., 2022a).

Similarly with previous results reported by Aly et al., we found that HLPF and SPF were independently associated with PLC injury (Aly et al., 2021, 2022b). Aly et al. also found significant independent associations between FJM and IDW and PLC injury (Aly et al., 2021). Although in our study we did not find an independent association, IDW was positively related with PLC injury, which might be explained by the interference of SPF when evaluating IDW (Hessmann et al., 2006). Although we found no collinearity between these two parameters, the measures of IDW might have been altered in CTs where SPF was also present, which might have underestimated the influence of IDW in our model. Along with the lack of consensus to define FJM, this parameter also had the lowest interrater reliability in our study, both of which might explain our results.

Based on recent literature, distinguishing between HLPF and VLPF when analyzing the diagnostic value of laminar fracture helped further strengthen the HLPF's associations with PLC injury, since VLPF has shown to not be independently associated with PLC injury in previous studies (Aly et al., 2021).

Khurana et al. and Aly et al., which found vertebral translation to be a significant predictor of PLC injury, since our goal was to create a score to help in decision-making, we excluded type C fractures, which have a high pretest probability of PLC injury, because independently of PLC status they will require surgical treatment (Khurana et al., 2018) (Aly et al., 2023).

When including all consensus CT measurements in a multivariate logistic regression model, we concluded that CTs reported as HLPF, SPF or IDW positive had significantly higher odds of PLC injury detected in MRI (7.66, 6.08 and 2.33, respectively). Although consistent with previous studies, all parameters proved to have lower odds than those previously reported (12.5, 16.2 and 4.4, respectively) (Aly et al., 2021).

Our best risk score (RS2) was able to estimate a total of 75.9% of correct classifications, with 71.0% of sensitivity and 78.3% of specificity. These results may appear less promising than previous ones. Aly et al. showed only 10% of misclassification using appropriate CT criteria to identify PLC injury (Aly et al., 2022b). However, this score strength relies on the different weights assigned to the parameters, based on their varied degrees of association (aOR) with PLC injury, rather than only counting each CT parameter as an equal finding. This approach may be closer to the real CT accuracy, yielding more generalizable results. Nevertheless, these results lack more evaluation and future analysis. When comparing the RS2 with the results from Aly et al., the CT parameters alone or combined were more reliable to predict PLC injury (Aly et al., 2022a). Our results reinforce the conclusions from previous studies about the importance of some CT parameters in helping clinicians to foresee nonobvious PLC lesion, without MRI (Aly et al., 2021, 2022b, 2023). Most importantly, our results reinforce the notion that the goal of CT will most certainly never be to replace the MRI, but to tendentially better infer the status of the PLC in scenarios were MRI is not a reality, while being a more accessible, economical, and faster exam. MRI. Overall, MRI should not be dispensed in patients with highly suggestive PLC injury.

### 4.1 Strengths and limitations

Our study has some limitations, including its retrospective, single-institution design. Inclusion of low-energy trauma cases may have introduced comorbidity bias, but enhanced comprehensiveness due to its growing relevance in the elderly (Tins, 2017). We did not include patients who were discharged based on negative CT findings without first undergoing MRI, thereby introducing verification bias (Khurana et al., 2018). The low number of cases with PLC injury (62) is probably the biggest limitation, in part due to the exclusion of patients with neurological deficits, because those cases constitute an independent reason to perform MRI and therefore surgical treatment. The main strength of this work is the study design and well-established methods such as the consecutive recruitment of patients with well-defined eligibility criteria, that helped minimizing selection bias (Aly et al., 2022a). We had four different raters, which is a larger number compared to previous studies (Ganjeifar et al., 2019; Aly et al., 2021, 2022a; Khurana et al., 2018; Aly et al., 2022a). Our investigators received consensus training to standardize the measurement and evaluation of each CT parameter (Aly et al., 2022a). However, we acknowledge that this reading might be less reproducible with reviewers with no previous training.

## 5 Conclusion

This study concludes that the standardized procedures pre-established in the CT measurement protocol were effective and can therefore be implemented. Overall, our study strengthened previous results reporting that CTs showing HLPF, SPF and IDW positive are significantly more likely to have PLC injury detected in MRI. Close attention to these three parameters may improve CT accuracy allowing it to play an important part in guiding treatment decisions in emergency settings in patients with lower pretest probability of PLC injury, when MRI is not available or when patients cannot undergo MRI.

## 6 Statements and declarations

No funds, grants, or other support was received. All authors certify that they have no affiliations with or involvement in any organization or entity with any financial interest or non-financial interest in the subject matter or materials discussed in this manuscript.
